# Application of Human Induced Pluripotent Stem Cell-Derived Cellular and Organoid Models for COVID-19 Research

**DOI:** 10.3389/fcell.2021.720099

**Published:** 2021-09-06

**Authors:** Yumei Luo, Mimi Zhang, Yapei Chen, Yaoyong Chen, Detu Zhu

**Affiliations:** ^1^Department of Obstetrics and Gynecology, Key Laboratory for Major Obstetric Diseases of Guangdong Province, The Third Affiliated Hospital of Guangzhou Medical University, Guangzhou, China; ^2^Key Laboratory of Reproduction and Genetics of Guangdong Higher Education Institutes, The Third Affiliated Hospital of Guangzhou Medical University, Guangzhou, China

**Keywords:** induced pluripotent stem cell, organoid, cellular model, COVID-19, SARS-CoV-2

## Abstract

The outbreak of severe acute respiratory syndrome coronavirus 2 (SARS-CoV-2) and its rapid international spread has caused the coronavirus disease 2019 (COVID-19) pandemics, which is a global public health crisis. Thus, there is an urgent need to establish biological models to study the pathology of SARS-CoV-2 infection, which not only involves respiratory failure, but also includes dysregulation of other organs and systems, including the brain, heart, liver, intestines, pancreas, kidneys, eyes, and so on. Cellular and organoid models derived from human induced pluripotent stem cells (iPSCs) are ideal tools for *in vitro* simulation of viral life cycles and drug screening to prevent the reemergence of coronavirus. These iPSC-derived models could recapitulate the functions and physiology of various human cell types and assemble the complex microenvironments similar with those in the human organs; therefore, they can improve the study efficiency of viral infection mechanisms, mimic the natural host-virus interaction, and be suited for long-term experiments. In this review, we focus on the application of *in vitro* iPSC-derived cellular and organoid models in COVID-19 studies.

## Introduction

Since its outbreak in 2019, the coronavirus disease (COVID-19) pandemics have infected more than 190 million people and caused more than 4 million deaths^1^. COVID-19 is caused by the severe acute respiratory syndrome coronavirus 2 (SARS-CoV-2), an enveloped positive-sense single-stranded RNA virus. It enters the host cells using angiotensin-converting enzyme 2 (ACE2) as the cell surface receptor and transmembrane serine protease 2 (TMPRSS2) as the effector to cleave its spike protein (Hoffmann et al., 2020). SARS-CoV-2 spreads mainly through the respiratory tract (Lu et al., 2020). Respiratory failure is the most common cause of death in COVID-19 patients; meanwhile, severe fatal manifestations are also observed in other organs, such as the brain, heart, liver, intestines, and pancreas (Puelles et al., 2020). Therefore, it is of particular importance to find models that can imitate the natural host-virus interactions of SARS-CoV-2 in a variety of human cell types and organs, thus improving the study efficiency for identifying key molecular regulators and the underlying mechanisms of virus infection and disease progression.

There have been both animal models (e.g., transgenic mice expressing human ACE2 and non-human primates) and cell line models (e.g., African green monkey Vero E6 cells and human cancer cell lines) available for COVID-19 research (Takayama, 2020). However, animal models are quite costly and display very different physiological characteristics from human, and cell line models have limitations in reproducing the viral life cycle and the pathology of COVID-19 in different human organs and tissues that contain a variety of cell types (Liu et al., 2011). For example, the entry routes of SARS-CoV-2 vary between cell lines and human tissues, as do immune responses and host-virus interactions (Milewska et al., 2020). In addition, human cancer cells carry numerous tumor-associated mutations, such as P53 mutations, which could interfere the SARS-CoV-2 infection (Ma-Lauer et al., 2016). Therefore, there is an urgent need to establish more cost-efficient and human-relevant models for COVID-19 research.

**FIGURE 1:**
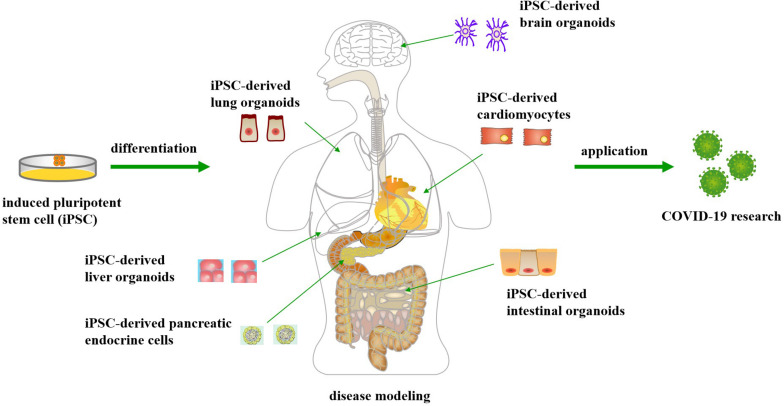
Application of human induced pluripotent stem cell-derived cellular and organoid models for COVID-19 research.

The emergence of human induced pluripotent stem cells (iPSCs) has enabled derivation of functional human cells or organoids to model human diseases, including infectious diseases, to develop new therapeutic approaches and to promote drug discovery (Yu et al., 2007; Luo et al., 2015a, 2018), without ethical issues like the human embryonic stem cells (Luo et al., 2014). For example, functional liver organoids generated by human iPSCs have been developed as personalized models of hepatitis B virus (HBV) infection, which is a powerful long-term platform for both research and drug screening of HBV (Nie et al., 2018). Recently, iPSC-derived cellular and organoid models have been utilized to simulate SARS-CoV-2 infections in multiple organs, not only the lung, but also the heart, brain, liver, intestines, and pancreas (Jacob et al., 2020; Yu et al., 2020) (Figure 1). These studies have demonstrated that SARS-CoV-2 can infect and propagate in a variety of cell types, leading to transcriptional alternations that indicate inflammatory responses and changes in cell function (Marshall, 2020). The purpose of this review is to describe the usefulness of these iPSC-derived cellular and organoid models in simulating human cellular physiology and tissue microenvironment, and enabling the study of host-virus interaction and drug screening for the COVID-19 disease, thus leading to a more comprehensive understanding of the SARS-CoV-2 pathogenesis.

## Lung Organoids

Human iPSC-derived airway and alveolar organoids have been developed and used for studying the processes of SARS-CoV-2 infection and transmission in the lungs (Pei et al., 2020). With these organoid models, the researchers have determined the cellular tropism of the virus. Ciliated cells, club cells, and alveolar type II cells (AT2) cells, which are arranged from the proximal to the distal airway and terminal alveoli in sequence, have been confirmed as SARS-CoV-2-targeted cells in the study (Mulay et al., 2021). Moreover, the viral infection downregulates metabolic processes, particularly the lipid metabolism, which, together with the already known upregulation of immune responses, is another molecular feature of SARS-CoV-2-infected cells (Pei et al., 2020). On the other hand, infected SARS-CoV-2 can decrease the level of its target ACE2 on the host cell surface through a variety of mechanisms. A comprehensive analysis of these information might enable better understanding about the viral pathogenesis and discovery therapeutic targets for the treatment of COVID-19 (Pei et al., 2020).

In addition, the early immune responses to viral infections were investigated using iPSC-derived AT2 (iAT2) cells (Huang J. et al., 2020). The results showed that AT2 cells are a central component of the inflammatory signaling that responds to SARS-CoV-2 infection within the first 24 h, with NF-κB signaling predominating this response (Huang J. et al., 2020). These findings are consistent with those in newly purified primary AT2 cells infected with SARS-CoV-2 (Mulay et al., 2021). They also observed cellular stress, toxicity, iAT cell death, and significant loss of surfactant genes expression in their model (Huang J. et al., 2020). These findings may be clinically relevant, as similar results were found in lung autopsies of multiple individuals who died of COVID-19 (Bradley et al., 2020; Hou et al., 2020). Other researchers have previously shown that the primary AT2 cells can be infected with SARS-CoV in the body (Qian et al., 2013); it has also been recently shown that AT2 cells may help promote lung regeneration in COVID-19 survivors (Chen J. et al., 2020). The correlation between AT2 cells and SARS-CoV-2 infection was further emphasized.

Furthermore, the researchers have demonstrated that human iPSC-derived lung cells and organoids could serve as powerful platforms for discovering and testing anti-SARS-CoV-2 drugs (Huang J. et al., 2020; Pei et al., 2020). They have demonstrated that Remdesivir, a predrug nucleotide analog that inhibits virus replication (Eastman et al., 2020), CB6, a human neutralizing antibody (Shi R. et al., 2020), as well as TMPRSS2 protease inhibition could effectively inhibit the replication of SARS-CoV-2 in the iPSC-derived models. These results are consistent with those in fundamental studies using primary cell models and in clinical trials (Beigel et al., 2020; Wang M. et al., 2020). Therefore, iPSC-derived *in vitro* human models could be employed to identify and test therapeutic entities for the treatment of COVID-19.

Taken together, the above results demonstrated that lung cells and organoids derived from human iPSCs can be utilized as pathophysiological models to study the potential mechanisms of SARS-CoV-2 transmission and to identify and test COVID-19 therapeutic agents (Pei et al., 2020).

## Cardiomyocytes

There is growing evidence that patients with COVID-19 exhibit severe heart complications, elevated biomarkers of heart damage, and cardiac function deterioration, including cardiovascular complications such as cardiomyopathy, acute myocardial infarction, arrhythmia, and heart failure, greatly increasing the risk of death (Aggarwal et al., 2020a; Bansal, 2020; Long et al., 2020; Madjid et al., 2020; Shi S. et al., 2020). Several compounds and antibodies, such as Remdesivir, Olumiant + Remdesivir, Casirivimab + Imdevimab, Bamlanivimab + Etesevimab, Sotrovimab and Tocilizumab are currently licensed drug for treatment of COVID-19 patients under emergency use authorization^2^. However, there is limited safety information on these recommended drugs, especially since heart toxicity caused by the drug can lead to lethal complications, including myocardial ischemia, arrhythmias, and heart failure. Therefore, it is critical to evaluate any potential adverse effects on the cardiovascular system associated with current COVID-19 medications to avoid fatal side effects (Aggarwal et al., 2020b).

Human iPSC-derived cardiomyocytes (CMs) can be utilized to recapitulate cardiac pathophysiology and are considered as one of the most promising sources for cardic disease modeling, heart repair and cardiac toxicology screening (Mitcheson et al., 1998; Lan et al., 2013; Moreno and Pearson, 2013; Sharma et al., 2014, 2017; Burridge et al., 2016). In this context, iPSC-CMs are recommended as a reliable method of heart toxicity examination in the comprehensive *in vitro* proarrhythmia assay (CiPA), as a non-clinical safety pharmacological paradigm, to circumvent the limitations of existing methods used in preclinical safety assessment of drugs (Gintant et al., 2016; Goineau and Castagné, 2017; Sala et al., 2017). Choi et al. (2020) have shown that iPSC-CMs acutely treated with Remdesivir show a risk of arrhythmia and changes in the electrophysiological properties of myocardial cells in a dose-dependent manner, indicating that overdose or drug accumulation may lead to noteworthy adverse heart reactions, such as prolonged QT interstitial periods. In addition, they have demonstrated that iPSC-CMs not only allow SARS-CoV-2 infection, but also support the propagation of infectious viral particles (Choi et al., 2020).

## Intestinal Organoids

Up to 50% of COVID-19 patients develop gastrointestinal symptoms associated with longer duration and increased severity of the disease (Luo et al., 2020; Wang F. et al., 2020; Wei et al., 2020; Xiao et al., 2020). However, it remains debatable whether the virus found in the intestines is contagious, as few studies have examined infectious viruses in feces (Zang et al., 2020). In cell culture, primary intestinal cells are highly susceptible to SARS-CoV-2 and can produce infectious viral particles. Intestinal organoids can quickly grow from adult stem cells derived from cells of large intestine and small intestine biopsy tissue (Sato et al., 2011). Stem cell-derived intestinal organoids have similar characteristics with primary intestinal cells and have been widely used to study viral infection (Sato et al., 2011; Ettayebi et al., 2016).

Studies have employed human iPSC-derived intestinal organoids to study SARS-CoV-2 tropisms in different intestinal cell types. In both *in vivo* and iPSC-derived organoid models, ACE2 is strongly expressed in the small intestine, as well as TMPRSS2. In contrast, colon organoids have lower ACE2 expression (Zang et al., 2020). In intestinal organs, TMPRSS4 performs the same functions as TMPRSS2 to support virus entry (Zang et al., 2020). Experiments have shown that two groups of small intestinal organoid models can be infected with SARS-CoV-2 (Lamers et al., 2020; Zang et al., 2020). In these organoid models, SARS-CoV-2, like its closest relative SARS-CoV, mainly infects mature enterocytes and dividing cells (Lamers et al., 2020; Zang et al., 2020). On the other hand, studies have shown that when the SARS-CoV-2 viruses are cultured in gastric fluid in the large intestine and small intestine, they quickly lose their infectious power (Simoneau and Ott, 2020; Zang et al., 2020). Therefore, even though viral particles were found in the feces occasionally, it might not be the primary pathway for virus transmission.

Furthermore, human iPSC-derived intestinal organoids generate valuable pathological models for studying the underlying mechanisms of intestinal SARS-CoV-2 infection. It is reported that SARS-CoV-2 actively infects both proximally and distally patterned intestinal organoids, thus resulting in production of infectious viral particles and significant transcriptional alterations, such as upregulation of the interferon-related genes, in multiple epithelial cell types (Mithal et al., 2021). Another organoid study shows that SARS-CoV-2 can infect all intestinal cell types investigated except goblet cells, and disrupt intestinal integrity, which might be the cause of diarrhea and other gastrointestinal symptoms associated with COVID-19 (Krüger et al., 2021).

More importantly, these organoids can serve as a potential platform for organ-specific drug testing and drug screening. For example, the researchers found that Remdesivir therapy inhibits SARS-CoV-2 viral replication in the intestinal organoids (Krüger et al., 2021). Therefore, clinical treatment with this drug may prevent intestinal damage caused by SARS-CoV-2 and relieve intestinal symptoms.

## Brain Organoids

Approximately 36.4% of the COVID-19 patients develop a variety of neurological complications, ranging from loss of smell, nausea, dizziness, and headache to encephalopathy and stroke (Mao et al., 2020). RNA of SARS-COV-2 was found in the brains of some patients (Helms et al., 2020; Moriguchi et al., 2020). The mechanisms of SARS-COV-2 disrupting the brain-blood barrier and infecting the central nervous system (CNS) draw great concerns (Li et al., 2020). Studies have shown that CNS infections may lead to the pathophysiological and clinical manifestations associated with COVID-19 (Steardo et al., 2020). Therefore, it is necessary to establish a suitable *in vitro* model to study nerve infection by SARS-CoV-2.

IPSC-derived brain organoids are valuable tools for investigating the biological properties of SARS-CoV-2 in the CNS (Bullen et al., 2020; Jacob et al., 2020). Studies using iPSC-derive brain organoids find that choroid plexus epithelial cells are the main target of SARS-CoV-2 infection in the CNS; meanwhile, neurons, and astrocytes are sparsely infected (Jacob et al., 2020; Pellegrini et al., 2020). This finding is consistent with the discovery that the choroid plexus region is one of the hotspots of ACE2 expression in the CNS under inflammatory status, and it is more susceptible to SARS-CoV-2 infection than other regions (Chen R. et al., 2020). After SARS-CoV-2 infection, increased cellular remodeling and inflammatory responses were observed in choroid plexus epithelial cells (Chen R. et al., 2020). This finding provides an evidence that SARS-CoV-2 infection of the choroid plexus leads to disruptions in blood-cerebrospinal fluid barrier (BCB) integrity. Researchers have proposed that BCB decomposition can promote entry of the virus as well as immune cells expressing cytokines into the cerebrospinal fluid and brain tissue, potentially causing nerve inflammation (Pellegrini et al., 2020).

Whether SARS-CoV-2 propagates in the CNS remains controversial. Some studies have reported successful SARS-CoV-2 replication in brain organoids (Zhang et al., 2020), while the others suggest that the viral replication and proliferation are less efficient in the brain organoids (Ramani et al., 2020). These opposite results may be due to differences in the methods of establishing brain organoid models and the multiplicities of infection (MOI) used in these studies. In the former study, the neural progenitor cell (NPC) population is also found to be a target of SARS-CoV-2 (Ramani et al., 2020). This is an important finding as NPCs are responsible for repairing brain lesions caused by degenerative diseases or malignancies (Zhu et al., 2013, 2014; Luo and Zhu, 2014; Luo et al., 2015b). The impaired NPC population might be the reason for late or incomplete recovery of neurological manifestations in COVID-19 patients. On the other hand, it should be noted that although human brain organoids represent valuable models for *in vitro* research on SARS-CoV-2 infection, they merely have simplified structures (e.g., vein systems and blood-brain barriers) like the developing fetal brain, and lack mature cells, particularly asteroid cells and astrocytes (Jacob et al., 2020; Ramani et al., 2020).

## Pancreatic Endocrine Cells

Single-cell RNA-seq analysis of primary human islets has indicated that both alpha cells and beta cells are positive for ACE2 and TMPRSS2 (Yang et al., 2020). Further validation experiments in humanized mouse model established by human iPSC-derived pancreatic endocrine cells confirm that both alpha cells and beta cells are susceptible to SARS-CoV-2 (Yang et al., 2020). Infected pancreatic endocrine cells display higher expression of pathways associated with apoptosis and viral infection, and lower expression of pathways associated with the normal functions of alpha cells and beta cells, thus leading to increased cell death and loss of cell identities (Yang et al., 2020). The infected cells are also expressing higher levels of chemokines, including CCL2, CXCL5, and CXCL6, and other degenerative factors and cytokines, which is similar with cells found in autopsies from COVID-19 patients (Blanco-Melo et al., 2020).

## Liver Organoids

More than 50% of COVID-19 patients have symptoms of viral hepatitis (Ong et al., 2020). Particularly, the proportion of patients with liver damage in patients with severe symptoms is much higher than that in patients with mild symptoms (Huang C. et al., 2020). However, due to the lack of suitable research models, it was unclear whether the liver damages were caused by a direct viral infection or by systemic dysfunctions, such as cytokine storms.

Relevant studies have deployed human organoids as tools to study the correlations of SARS-CoV-2 infection and liver damage at both cellular and molecular levels (Huch et al., 2015; Dutta and Clevers, 2017). Some studies have established human liver organoid models which are capable of preserving the ACE2 + /TMPRSS2 + cholangiocyte population in long-term 3-dimensional (3D) cultures (Yang et al., 2020; Zhao et al., 2020). Further, the studies have confirmed that the cholangiocytes in the human liver organoids are permissive to SARS-CoV-2 infection and supporting strong viral propagation (Yang et al., 2020; Zhao et al., 2020). Moreover, SARS-CoV-2 infection induces cell death in the host cholangiocytes. Thus, these studies supports that liver damage in COVID-19 patients might be caused by gallbladder decomposition and subsequent accumulation of bile acid due to viral infection (Yang et al., 2020; Zhao et al., 2020).

## Discussion and Future Perspectives

Most COVID-19 patients have mild respiratory symptoms; however, up to 20% of the patients develop severe pneumonia, leading to multi-organ failures and even death (Zhu N. et al., 2020). The development of iPSC technologies and the resulting differentiated cell models have dramatically accelerated studies of the pathogenesis of SARS-CoV-2 in various organs. Current studies have employed iPSC-derived cells and organoids, including iAT2 cells, cardiomyocytes, pancreatic endocrine cells, lung organoids, brain organoids, intestinal organoids, liver organoids, to investigate the underlying mechanisms of SARS-CoV-2 infection (Ardestani and Maedler, 2020; Bojkova et al., 2020; Huang J. et al., 2020; Jacob et al., 2020; Pei et al., 2020; Mithal et al., 2021). As COVID-19 could also cause kidney malfunctions (Chen N. et al., 2020), kidney organoids derived from iPSCs may be a potential research model as well (Phipson et al., 2019).

These iPSC-derived models are suited for leveraging the powers of the latest genetic tools, such as single-cell RNA-seq and CRISPR techniques, for COVID-19 research (Zhou et al., 2020). Single-cell RNA-seq techniques have been developed to investigate the viral tropisms and host transcriptional responses to viruses or external stimuli in complex organs and tissues (Zhu et al., 2018; Luo et al., 2019; Zhu D. et al., 2020). In the above studies, single-cell RNA-seq has been employed to screen for cell types that are positive of the SARS-CoV-2 receptor ACE2 and effector protease TMPRSS2, and to illustrate the transcriptional alternations after viral infection (Huang J. et al., 2020; Yang et al., 2020; Zang et al., 2020). Furthermore, the CRISPR system can be utilized to create genetically modified iPSC models for mechanism study of the candidate genetic factors (Luo et al., 2015c, 2016; Gkogkou et al., 2020; Kim et al., 2020; Zang et al., 2020; Yu, 2021). For example, with a CRISPR-engineered iPSC model, researchers have demonstrated that the single-nucleotide polymorphism rs4702, which is a common genetic variant located in the 3′ UTR of the protease FURIN, influences the SARS-CoV-2 permissiveness of alveolar and neuronal cells (Dobrindt et al., 2021).

On the other hand, there are a few limitations of iPSC-derived cellular and organoid platforms, such as inadequate complexity to reflect real tissue microenvironment and cell-cell interactions, and lack of real-time monitoring methods for 3D cultures. For examples, although the above studies have described the susceptibility of various cell types to SARS-CoV-2 infection with these iPSC derivatives, it is unclear whether these cell types are the primary targets for viral infection in COVID-19 without a more thorough analysis of samples from primary patients (Lamouroux et al., 2020). Besides, these iPSC-derived models are simplified ones compared to the fully functional and reacting human organs. In the future, these platforms should be exploited to produce more complex organoid models, including the immune system components that are missing from the current analysis (Yang et al., 2020).

## Conclusion

In conclusion, human iPSC-derived cells and organoids can be used as ideal models for studying the mechanisms of viral infection and drug screening. Particularly, with the organoid models, the viral tropism and host responses of different cell types could be observed in a single system. These iPSC models help us better understand the pathogenesis of SARS-CoV-2 in different organs and systems, and provide powerful drug test and discovery platforms.

## Author Contributions

YL and DZ conceived the study. YL, MZ, and DZ prepared the figure. YL, MZ, YpC, YoC, and DZ wrote the manuscript. All authors read and approved the final manuscript.

## Conflict of Interest

The authors declare that the research was conducted in the absence of any commercial or financial relationships that could be construed as a potential conflict of interest.

## Publisher’s Note

All claims expressed in this article are solely those of the authors and do not necessarily represent those of their affiliated organizations, or those of the publisher, the editors and the reviewers. Any product that may be evaluated in this article, or claim that may be made by its manufacturer, is not guaranteed or endorsed by the publisher.

## Footnotes

1
www.worldometers.info/coronavirus

2
www.fda.gov/emergency-preparedness-and-response/mcm-legal-regulatory-and-policy-framework/emergency-use-authorization#coviddrugs

## References

[B1] Aggarwal G. Cheruiyot I. Aggarwal S. Wong J. Lippi G. Lavie C. J. (2020a). Association of Cardiovascular Disease With Coronavirus Disease 2019 (COVID-19) Severity: a Meta-Analysis. *Curr. Probl. Cardiol.* 45:100617. 10.1016/j.cpcardiol.2020.100617 32402515 PMC7187816

[B2] Aggarwal G. Henry B. M. Aggarwal S. Bangalore S. (2020b). Cardiovascular Safety of Potential Drugs for the Treatment of Coronavirus Disease 2019. *Am. J. Cardiol.* 128 147–150. 10.1016/j.amjcard.2020.04.054 32425199 PMC7228893

[B3] Ardestani A. Maedler K. (2020). Commentary: a Human Pluripotent Stem Cell-Based Platform to Study SARS-CoV-2 Tropism and Model Virus Infection in Human Cells and Organoids. *Front. Endocrinol.* 11:585922. 10.3389/fendo.2020.585922 33162939 PMC7591701

[B4] Bansal M. (2020). Cardiovascular disease and COVID-19. *Diabetes Metab. Syndr.* 14 247–250. 32247212 10.1016/j.dsx.2020.03.013 PMC7102662

[B5] Beigel J. H. Tomashek K. M. Dodd L. E. Mehta A. K. Zingman B. S. Kalil A. C. (2020). Remdesivir for the Treatment of Covid-19 - Final Report. *N. Engl. J. Med.* 383 1813–1826. 32445440 10.1056/NEJMoa2007764 PMC7262788

[B6] Blanco-Melo D. Nilsson-Payant B. E. Liu W. C. Uhl S. Hoagland D. Møller R. (2020). Imbalanced Host Response to SARS-CoV-2 Drives Development of COVID-19. *Cell* 181 1036–1045.e9. 32416070 10.1016/j.cell.2020.04.026 PMC7227586

[B7] Bojkova D. Wagner J. U. G. Shumliakivska M. Aslan G. S. Saleem U. Hansen A. (2020). SARS-CoV-2 infects and induces cytotoxic effects in human cardiomyocytes. *Cardiovasc. Res.* 116 2207–2215. 10.1093/cvr/cvaa267 32966582 PMC7543363

[B8] Bradley B. T. Maioli H. Johnston R. Chaudhry I. Fink S. L. Xu H. (2020). Histopathology and ultrastructural findings of fatal COVID-19 infections in Washington State: a case series. *Lancet* 396 320–332. 10.1016/s0140-6736(20)31305-2 32682491 PMC7365650

[B9] Bullen C. K. Hogberg H. T. Bahadirli-Talbott A. Bishai W. R. Hartung T. Keuthan C. (2020). Infectability of human BrainSphere neurons suggests neurotropism of SARS-CoV-2. *Altex* 37 665–671. 32591839 10.14573/altex.2006111

[B10] Burridge P. W. Li Y. F. Matsa E. Wu H. Ong S. G. Sharma A. (2016). Human induced pluripotent stem cell-derived cardiomyocytes recapitulate the predilection of breast cancer patients to doxorubicin-induced cardiotoxicity. *Nat. Med.* 22 547–556. 10.1038/nm.4087 27089514 PMC5086256

[B11] Chen J. Wu H. Yu Y. Tang N. (2020). Pulmonary alveolar regeneration in adult COVID-19 patients. *Cell Res.* 30 708–710. 10.1038/s41422-020-0369-7 32632255 PMC7338112

[B12] Chen N. Zhou M. Dong X. Qu J. Gong F. Han Y. (2020). Epidemiological and clinical characteristics of 99 cases of 2019 novel coronavirus pneumonia in Wuhan, China: a descriptive study. *Lancet* 395 507–513. 10.1016/s0140-6736(20)30211-7 32007143 PMC7135076

[B13] Chen R. Wang K. Yu J. Howard D. French L. Chen Z. (2020). The Spatial and Cell-Type Distribution of SARS-CoV-2 Receptor ACE2 in the Human and Mouse Brains. *Front. Neurol.* 11:573095. 10.3389/fneur.2020.573095 33551947 PMC7855591

[B14] Choi S. W. Shin J. S. Park S. J. Jung E. Park Y. G. Lee J. (2020). Antiviral activity and safety of remdesivir against SARS-CoV-2 infection in human pluripotent stem cell-derived cardiomyocytes. *Antiviral Res.* 184:104955. 10.1016/j.antiviral.2020.104955 33091434 PMC7571425

[B15] Dobrindt K. Hoagland D. A. Seah C. Kassim B. O’Shea C. P. Murphy A. (2021). Common Genetic Variation in Humans Impacts In Vitro Susceptibility to SARS-CoV-2 Infection. *Stem Cell Rep.* 16 505–518. 10.1016/j.stemcr.2021.02.010 33636110 PMC7881728

[B16] Dutta D. Clevers H. (2017). Organoid culture systems to study host-pathogen interactions. *Curr. Opin. Immunol.* 48 15–22. 10.1016/j.coi.2017.07.012 28756233 PMC7126332

[B17] Eastman R. T. Roth J. S. Brimacombe K. R. Simeonov A. Shen M. Patnaik S. (2020). Remdesivir: a Review of Its Discovery and Development Leading to Emergency Use Authorization for Treatment of COVID-19. *ACS Cent. Sci.* 6 672–683. 10.1021/acscentsci.0c00489 32483554 PMC7202249

[B18] Ettayebi K. Crawford S. E. Murakami K. Broughman J. R. Karandikar U. Tenge V. R. (2016). Replication of human noroviruses in stem cell-derived human enteroids. *Science* 353 1387–1393. 10.1126/science.aaf5211 27562956 PMC5305121

[B19] Gintant G. Sager P. T. Stockbridge N. (2016). Evolution of strategies to improve preclinical cardiac safety testing. *Nat. Rev. Drug Discov.* 15 457–471. 10.1038/nrd.2015.34 26893184

[B20] Gkogkou E. Barnasas G. Vougas K. Trougakos I. P. (2020). Expression profiling meta-analysis of ACE2 and TMPRSS2, the putative anti-inflammatory receptor and priming protease of SARS-CoV-2 in human cells, and identification of putative modulators. *Redox Biol.* 36:101615. 10.1016/j.redox.2020.101615 32863223 PMC7311357

[B21] Goineau S. Castagné V. (2017). Proarrhythmic risk assessment using conventional and new in vitro assays. *Regul. Toxicol. Pharmacol.* 88 1–11. 10.1016/j.yrtph.2017.05.012 28506844

[B22] Helms J. Kremer S. Merdji H. Clere-Jehl R. Schenck M. Kummerlen C. (2020). Neurologic Features in Severe SARS-CoV-2 Infection. *N. Engl. J. Med.* 382 2268–2270. 32294339 10.1056/NEJMc2008597 PMC7179967

[B23] Hoffmann M. Kleine-Weber H. Schroeder S. Krüger N. Herrler T. Erichsen S. (2020). Drosten and S. Pöhlmann, SARS-CoV-2 Cell Entry Depends on ACE2 and TMPRSS2 and Is Blocked by a Clinically Proven Protease Inhibitor. *Cell* 181 271–280.e8. 32142651 10.1016/j.cell.2020.02.052 PMC7102627

[B24] Hou Y. J. Okuda K. Edwards C. E. Martinez D. R. Asakura T. Dinnon K. H. III (2020). SARS-CoV-2 Reverse Genetics Reveals a Variable Infection Gradient in the Respiratory Tract. *Cell* 182 429–446.e14. 32526206 10.1016/j.cell.2020.05.042 PMC7250779

[B25] Huang C. Wang Y. Li X. Ren L. Zhao J. Hu Y. (2020). Clinical features of patients infected with 2019 novel coronavirus in Wuhan, China. *Lancet* 395 497–506. 31986264 10.1016/S0140-6736(20)30183-5 PMC7159299

[B26] Huang J. Hume A. J. Abo K. M. Werder R. B. Villacorta-Martin C. Alysandratos K. D. (2020). SARS-CoV-2 Infection of Pluripotent Stem Cell-Derived Human Lung Alveolar Type 2 Cells Elicits a Rapid Epithelial-Intrinsic Inflammatory Response. *Cell Stem Cell* 27 962–973.e7. 32979316 10.1016/j.stem.2020.09.013 PMC7500949

[B27] Huch M. Gehart H. van Boxtel R. Hamer K. Blokzijl F. Verstegen M. M. (2015). Long-term culture of genome-stable bipotent stem cells from adult human liver. *Cell* 160 299–312. 10.1016/j.cell.2014.11.050 25533785 PMC4313365

[B28] Jacob F. Pather S. R. Huang W. K. Zhang F. Wong S. Z. H. Zhou H. (2020). Human Pluripotent Stem Cell-Derived Neural Cells and Brain Organoids Reveal SARS-CoV-2 Neurotropism Predominates in Choroid Plexus Epithelium. *Cell Stem Cell* 27 937–950.e9. 33010822 10.1016/j.stem.2020.09.016 PMC7505550

[B29] Kim J. Koo B. K. Knoblich J. A. (2020). Human organoids: model systems for human biology and medicine. *Nat. Rev. Mol. Cell Biol.* 21 571–584. 10.1038/s41580-020-0259-3 32636524 PMC7339799

[B30] Krüger J. Groß R. Conzelmann C. Müller J. A. Koepke L. Sparrer K. M. J. (2021). Drug Inhibition of SARS-CoV-2 Replication in Human Pluripotent Stem Cell-Derived Intestinal Organoids. *Cell. Mol. Gastroenterol. Hepatol.* 11 935–948. 10.1016/j.jcmgh.2020.11.003 33186749 PMC7655023

[B31] Lamers M. M. Beumer J. van der Vaart J. Knoops K. Puschhof J. Breugem T. I. (2020). SARS-CoV-2 productively infects human gut enterocytes. *Science* 369 50–54. 10.1126/science.abc1669 32358202 PMC7199907

[B32] Lamouroux A. Attie-Bitach T. Martinovic J. Leruez-Ville M. Ville Y. (2020). Evidence for and against vertical transmission for severe acute respiratory syndrome coronavirus 2. *Am. J. Obstet. Gynecol.* 223 91.e1–91.e4. 32376317 10.1016/j.ajog.2020.04.039 PMC7196550

[B33] Lan F. Lee A. S. Liang P. Sanchez-Freire V. Nguyen P. K. Wang L. (2013). Abnormal calcium handling properties underlie familial hypertrophic cardiomyopathy pathology in patient-specific induced pluripotent stem cells. *Cell Stem Cell* 12 101–113. 10.1016/j.stem.2012.10.010 23290139 PMC3638033

[B34] Li Y. C. Bai W. Z. Hashikawa T. (2020). The neuroinvasive potential of SARS-CoV2 may play a role in the respiratory failure of COVID-19 patients. *J. Med. Virol.* 92 552–555. 10.1002/jmv.25728 32104915 PMC7228394

[B35] Liu L. Wei Q. Alvarez X. Wang H. Du Y. Zhu H. (2011). Epithelial cells lining salivary gland ducts are early target cells of severe acute respiratory syndrome coronavirus infection in the upper respiratory tracts of rhesus macaques. *J. Virol.* 85 4025–4030. 10.1128/jvi.02292-10 21289121 PMC3126125

[B36] Long B. Brady W. J. Koyfman A. Gottlieb M. (2020). Cardiovascular complications in COVID-19. *Am. J. Emerg. Med.* 38 1504–1507. 32317203 10.1016/j.ajem.2020.04.048 PMC7165109

[B37] Lu R. Zhao X. Li J. Niu P. Yang B. Wu H. (2020). Genomic characterisation and epidemiology of 2019 novel coronavirus: implications for virus origins and receptor binding. *Lancet* 395 565–574. 10.1016/s0140-6736(20)30251-8 32007145 PMC7159086

[B38] Luo S. Zhang X. Xu H. (2020). Don’t Overlook Digestive Symptoms in Patients With 2019 Novel Coronavirus Disease (COVID-19). *Clin. Gastroenterol. Hepatol.* 18 1636–1637. 10.1016/j.cgh.2020.03.043 32205220 PMC7154217

[B39] Luo Y. Huang J. Tang Y. Luo X. Ge L. Sheng X. (2019). Regional methylome profiling reveals dynamic epigenetic heterogeneity and convergent hypomethylation of stem cell quiescence-associated genes in breast cancer following neoadjuvant chemotherapy. *Cell Biosci.* 9:16. 10.1186/s13578-019-0278-y PMC6367786 30774927

[B40] Luo Y. Li J. Zhu D. Fan Y. Li S. Sun X. (2014). High-resolution chromosomal microarray analysis of early-stage human embryonic stem cells reveals an association between X chromosome instability and skewed X inactivation. *Cell Biosci* 4:74. 10.1186/2045-3701-4-74 25506417 PMC4265433

[B41] Luo Y. Xu X. An X. Sun X. Wang S. Zhu D. (2016). Targeted Inhibition of the miR-199a/214 Cluster by CRISPR Interference Augments the Tumor Tropism of Human Induced Pluripotent Stem Cell-Derived Neural Stem Cells under Hypoxic Condition. *Stem Cells Int.* 2016:3598542. 10.1155/2016/3598542 PMC5124688 27965712

[B42] Luo Y. Zhu D. (2014). Combinatorial control of transgene expression by hypoxia-responsive promoter and microrna regulation for neural stem cell-based cancer therapy. *Biomed. Res. Int.* 2014:751397. 10.1155/2014/751397 PMC4016878 24864258

[B43] Luo Y. Zhu D. Du R. Gong Y. Xie C. Xu X. (2015a). Uniparental disomy of the entire X chromosome in Turner syndrome patient-specific induced pluripotent stem cells. *Cell Discov.* 1:15022. 10.1038/celldisc.2015.22 PMC4860828 27462421

[B44] Luo Y. Zhu D. Lam D. H. Huang J. Tang Y. Luo X. (2015b). A Double-Switch Cell Fusion-Inducible Transgene Expression System for Neural Stem Cell-Based Antiglioma Gene Therapy. *Stem Cells Int.* 2015:649080. 10.1155/2015/649080 PMC4436471 26074975

[B45] Luo Y. Zhu D. Xu X. Ge L. Sun X. Chen G. (2018). Generation of an induced pluripotent stem cell line from an adult male with 45,X/46,XY mosaicism. *Stem Cell Res* 27 42–45. 10.1016/j.scr.2018.01.003 29320756

[B46] Luo Y. Zhu D. Zhang Z. Chen Y. Sun X. (2015c). Integrative Analysis of CRISPR/Cas9 Target Sites in the Human HBB Gene. *Biomed. Res. Int.* 2015:514709. 10.1155/2015/514709 PMC4396065 25918715

[B47] Madjid M. Safavi-Naeini P. Solomon S. D. Vardeny O. (2020). Potential Effects of Coronaviruses on the Cardiovascular System: a Review. *JAMA Cardiol.* 5 831–840. 10.1001/jamacardio.2020.1286 32219363

[B48] Ma-Lauer Y. Carbajo-Lozoya J. Hein M. Y. Müller M. A. Deng W. Lei J. (2016). p53 down-regulates SARS coronavirus replication and is targeted by the SARS-unique domain and PLpro via E3 ubiquitin ligase RCHY1. *Proc. Natl. Acad. Sci. U. S. A.* 113 E5192–E5201. 27519799 10.1073/pnas.1603435113 PMC5024628

[B49] Mao L. Jin H. Wang M. Hu Y. Chen S. He Q. (2020). Neurologic Manifestations of Hospitalized Patients With Coronavirus Disease 2019 in Wuhan, China. *JAMA Neurol.* 77 683–690. 10.1001/jamaneurol.2020.1127 32275288 PMC7149362

[B50] Marshall M. (2020). The lasting misery of coronavirus long-haulers. *Nature* 585 339–341. 10.1038/d41586-020-02598-6 32929257

[B51] Milewska A. Kula-Pacurar A. Wadas J. Suder A. Szczepanski A. Dabrowska A. (2020). Replication of Severe Acute Respiratory Syndrome Coronavirus 2 in Human Respiratory Epithelium. *J. Virol.* 94 e00957–20. 32434888 10.1128/JVI.00957-20 PMC7375387

[B52] Mitcheson J. S. Hancox J. C. Levi A. J. (1998). Cultured adult cardiac myocytes: future applications, culture methods, morphological and electrophysiological properties. *Cardiovasc. Res.* 39 280–300. 10.1016/s0008-6363(98)00128-x 9798514

[B53] Mithal A. Hume A. J. Lindstrom-Vautrin J. Villacorta-Martin C. Olejnik J. Bullitt E. (2021). Human Pluripotent Stem Cell-Derived Intestinal Organoids Model SARS-CoV-2 Infection Revealing a Common Epithelial Inflammatory Response. *Stem Cell Rep.* 16 940–953. 10.1016/j.stemcr.2021.02.019 33852884 PMC8042780

[B54] Moreno L. Pearson A. D. (2013). How can attrition rates be reduced in cancer drug discovery? *Expert. Opin. Drug Discov.* 8 363–368. 10.1517/17460441.2013.768984 23373702

[B55] Moriguchi T. Harii N. Goto J. Harada D. Sugawara H. Takamino J. (2020). A first case of meningitis/encephalitis associated with SARS-Coronavirus-2. *Int. J. Infect. Dis.* 94 55–58. 32251791 10.1016/j.ijid.2020.03.062 PMC7195378

[B56] Mulay A. Konda B. Garcia G. Jr. Yao C. Beil S. Villalba J. M. (2021). SARS-CoV-2 infection of primary human lung epithelium for COVID-19 modeling and drug discovery. *Cell Rep.* 35:109055. 10.1016/j.celrep.2021.109055 33905739 PMC8043574

[B57] Nie Y. Z. Zheng Y. W. Miyakawa K. Murata S. Zhang R. R. Sekine K. (2018). Recapitulation of hepatitis B virus-host interactions in liver organoids from human induced pluripotent stem cells. *EBioMedicine* 35 114–123. 10.1016/j.ebiom.2018.08.014 30120080 PMC6156717

[B58] Ong J. Young B. E. Ong S. (2020). COVID-19 in gastroenterology: a clinical perspective. *Gut* 69 1144–1145. 10.1136/gutjnl-2020-321051 32198152

[B59] Pei R. Feng J. Zhang Y. Sun H. Li L. Yang X. (2020). Host metabolism dysregulation and cell tropism identification in human airway and alveolar organoids upon SARS-CoV-2 infection. *Protein Cell* [Epub Online ahead of print]. 10.1007/s13238-020-00811-w 33314005 PMC7732737

[B60] Pellegrini L. Albecka A. Mallery D. L. Kellner M. J. Paul D. Carter A. P. (2020). SARS-CoV-2 Infects the Brain Choroid Plexus and Disrupts the Blood-CSF Barrier in Human Brain Organoids. *Cell Stem Cell* 27 951–961.e5. 33113348 10.1016/j.stem.2020.10.001 PMC7553118

[B61] Phipson B. Er P. X. Combes A. N. Forbes T. A. Howden S. E. Zappia L. (2019). Evaluation of variability in human kidney organoids. *Nat. Methods* 16 79–87. 10.1038/s41592-018-0253-2 30573816 PMC6634992

[B62] Puelles V. G. Lütgehetmann M. Lindenmeyer M. T. Sperhake J. P. Wong M. N. Allweiss L. (2020). Multiorgan and Renal Tropism of SARS-CoV-2. *N. Engl. J. Med.* 383 590–592. 32402155 10.1056/NEJMc2011400 PMC7240771

[B63] Qian Z. Travanty E. A. Oko L. Edeen K. Berglund A. Wang J. (2013). Innate immune response of human alveolar type II cells infected with severe acute respiratory syndrome-coronavirus. *Am. J. Respir. Cell Mol. Biol.* 48 742–748. 10.1165/rcmb.2012-0339oc 23418343 PMC3727876

[B64] Ramani A. Müller L. Ostermann P. N. Gabriel E. Abida-Islam P. Müller-Schiffmann A. (2020). SARS-CoV-2 targets neurons of 3D human brain organoids. *EMBO J.* 39:e106230. 10.15252/embj.2020106230 PMC7560208 32876341

[B65] Sala L. Bellin M. Mummery C. L. (2017). Integrating cardiomyocytes from human pluripotent stem cells in safety pharmacology: has the time come? *Br. J. Pharmacol.* 174 3749–3765. 10.1111/bph.13577 27641943 PMC5647193

[B66] Sato T. Stange D. E. Ferrante M. Vries R. G. Van Es J. H. Van den Brink S. (2011). Long-term expansion of epithelial organoids from human colon, adenoma, adenocarcinoma, and Barrett’s epithelium. *Gastroenterology* 141 1762–1772. 10.1053/j.gastro.2011.07.050 21889923

[B67] Sharma A. Burridge P. W. McKeithan W. L. Serrano R. Shukla P. Sayed N. (2017). High-throughput screening of tyrosine kinase inhibitor cardiotoxicity with human induced pluripotent stem cells. *Sci. Transl. Med.* 9:eaaf2584. 10.1126/scitranslmed.aaf2584 28202772 PMC5409837

[B68] Sharma A. Marceau C. Hamaguchi R. Burridge P. W. Rajarajan K. Churko J. M. (2014). Human induced pluripotent stem cell-derived cardiomyocytes as an in vitro model for coxsackievirus B3-induced myocarditis and antiviral drug screening platform. *Circ. Res.* 115 556–566. 10.1161/circresaha.115.303810 25015077 PMC4149868

[B69] Shi R. Shan C. Duan X. Chen Z. Liu P. Song J. (2020). A human neutralizing antibody targets the receptor-binding site of SARS-CoV-2. *Nature* 584 120–124. 32454512 10.1038/s41586-020-2381-y

[B70] Shi S. Qin M. Shen B. Cai Y. Liu T. Yang F. (2020). Association of Cardiac Injury With Mortality in Hospitalized Patients With COVID-19 in Wuhan, China. *JAMA Cardiol.* 5 802–810. 10.1001/jamacardio.2020.0950 32211816 PMC7097841

[B71] Simoneau C. R. Ott M. (2020). Modeling Multi-organ Infection by SARS-CoV-2 Using Stem Cell Technology. *Cell Stem Cell* 27 859–868. 10.1016/j.stem.2020.11.012 33275899 PMC7713543

[B72] Steardo L. Steardo L. Jr. Zorec R. Verkhratsky A. (2020). Neuroinfection may contribute to pathophysiology and clinical manifestations of COVID-19. *Acta Physiol.* 229:e13473. 10.1111/apha.13473 PMC7228251 32223077

[B73] Takayama K. (2020). In Vitro and Animal Models for SARS-CoV-2 research. *Trends Pharmacol. Sci.* 41 513–517. 10.1016/j.tips.2020.05.005 32553545 PMC7260555

[B74] Wang F. Wang H. Fan J. Zhang Y. Wang H. Zhao Q. (2020). Pancreatic Injury Patterns in Patients With Coronavirus Disease 19 Pneumonia. *Gastroenterology* 159 367–370. 10.1053/j.gastro.2020.03.055 32247022 PMC7118654

[B75] Wang M. Cao R. Zhang L. Yang X. Liu J. Xu M. (2020). Remdesivir and chloroquine effectively inhibit the recently emerged novel coronavirus (2019-nCoV) in vitro. *Cell Res.* 30 269–271. 10.1038/s41422-020-0282-0 32020029 PMC7054408

[B76] Wei X. S. Wang X. Niu Y. R. Ye L. L. Peng W. B. Wang Z. H. (2020). Diarrhea Is Associated With Prolonged Symptoms and Viral Carriage in Corona Virus Disease 2019. *Clin. Gastroenterol. Hepatol.* 18 1753–1759.e2. 32311512 10.1016/j.cgh.2020.04.030 PMC7165091

[B77] Xiao F. Tang M. Zheng X. Liu Y. Li X. Shan H. (2020). Evidence for Gastrointestinal Infection of SARS-CoV-2. *Gastroenterology* 158 1831–1833.e3. 32142773 10.1053/j.gastro.2020.02.055 PMC7130181

[B78] Yang L. Han Y. Nilsson-Payant B. E. Gupta V. Wang P. Duan X. (2020). A *Human Pluripotent Stem Cell-based Platform to Study SARS-CoV-2 Tropism and Model Virus Infection in Human Cells and Organoids*. *Cell Stem Cell* 27 125–136.e7. 32579880 10.1016/j.stem.2020.06.015 PMC7303620

[B79] Yu F. Jia R. Tang Y. Liu J. Wei B. (2020). SARS-CoV-2 infection and stem cells: interaction and intervention. *Stem Cell Res.* 46:101859. 10.1016/j.scr.2020.101859 32570174 PMC7263221

[B80] Yu J. (2021). Organoids: a New Model for SARS-CoV-2 Translational Research. *Int. J. Stem Cells* 14 138–149. 10.15283/ijsc20169 33632991 PMC8138661

[B81] Yu J. Vodyanik M. A. Smuga-Otto K. Antosiewicz-Bourget J. Frane J. L. Tian S. (2007). Induced pluripotent stem cell lines derived from human somatic cells. *Science* 318 1917–1920. 18029452 10.1126/science.1151526

[B82] Zang R. Castro M.F. Gomez McCune B. T. Zeng Q. Rothlauf P. W. Sonnek N. M. (2020). TMPRSS2 and TMPRSS4 promote SARS-CoV-2 infection of human small intestinal enterocytes. *Sci. Immunol.* 5:eabc3582. 10.1126/sciimmunol.abc3582 32404436 PMC7285829

[B83] Zhang B. Z. Chu H. Han S. Shuai H. Deng J. Hu Y. F. (2020). SARS-CoV-2 infects human neural progenitor cells and brain organoids. *Cell Res.* 30 928–931. 10.1038/s41422-020-0390-x 32753756 PMC7399356

[B84] Zhao B. Ni C. Gao R. Wang Y. Yang L. Wei J. (2020). Recapitulation of SARS-CoV-2 infection and cholangiocyte damage with human liver ductal organoids. *Protein Cell* 11 771–775. 32303993 10.1007/s13238-020-00718-6 PMC7164704

[B85] Zhou H. Liu L. P. Fang M. Li Y. M. Zheng Y. W. (2020). A potential ex vivo infection model of human induced pluripotent stem cell-3D organoids beyond coronavirus disease 2019. *Histol. Histopathol.* 35 1077–1082. 32339250 10.14670/HH-18-223

[B86] Zhu D. Chen C. Purwanti Y. I. Du S. Lam D. H. Wu C. (2014). Induced pluripotent stem cell-derived neural stem cells transduced with baculovirus encoding CD40 ligand for immunogene therapy in mouse models of breast cancer. *Hum. Gene Ther.* 25 747–758. 10.1089/hum.2013.160 24773154

[B87] Zhu D. Lam D. H. Purwanti Y. I. Goh S. L. Wu C. Zeng J. (2013). Systemic delivery of fusogenic membrane glycoprotein-expressing neural stem cells to selectively kill tumor cells. *Mol. Ther.* 21 1621–1630. 10.1038/mt.2013.123 23752308 PMC3734663

[B88] Zhu D. Rostami M. R. Zuo W. L. Leopold P. L. Crystal R. G. (2020). Single-Cell Transcriptome Analysis of Mouse Liver Cell-Specific Tropism and Transcriptional Dysregulation Following Intravenous Administration of AAVrh.10 Vectors. *Hum. Gene Ther.* 31 590–604. 10.1089/hum.2019.366 32143547 PMC7232697

[B89] Zhu D. Zhao Z. Cui G. Chang S. Hu L. See Y. X. (2018). Single-Cell Transcriptome Analysis Reveals Estrogen Signaling Coordinately Augments One-Carbon, Polyamine, and Purine Synthesis in Breast Cancer. *Cell Rep.* 25 2285–2298.e4. 30463022 10.1016/j.celrep.2018.10.093

[B90] Zhu N. Zhang D. Wang W. Li X. Yang B. Song J. (2020). A Novel Coronavirus from Patients with Pneumonia in China, 2019. *N. Engl. J. Med.* 382 727–733. 31978945 10.1056/NEJMoa2001017 PMC7092803

